# Ways to improve guideline adherence in the emergency department: an interview study on the management of traumatic brain injuries

**DOI:** 10.1007/s00068-021-01853-3

**Published:** 2022-02-03

**Authors:** Sebastian Vestlund, Tomas Vedin, Marcus Edelhamre, Magnus Lindén, Per-Anders Larsson

**Affiliations:** 1 Department of Clinical Sciences, Faculty of Medicine, Lund University, Lund, Sweden; 2 Department of Psychology, Faculty of Social Sciences, Lund University, Lund, Sweden; 3 Department of Surgery, Skaraborg Hospital, Skövde, Sweden

**Keywords:** Guideline adherence, Emergency department, Traumatic brain injuries, Decision rules

## Abstract

**Purpose:**

The aim was to explore factors affecting guideline adherence among doctors in the emergency department and to explore the general perception about local guidelines for traumatic brain injuries.

**Methods:**

Thirty semi-structured interviews were conducted with doctors with experience working in the emergency department regarding different aspects of guideline use, with emphasis on the management of traumatic brain injuries. Twenty-eight interviews were included for analysis. The interviews were recorded, transcribed, and analysed iteratively. Emergent codes were identified and organised into themes and subthemes.

**Results:**

Eight themes were identified. Barriers were centred on low availability of local guidelines and guideline document design. Facilitating factors included a concise document, appropriate visual aids, high accessibility, and encouragement by management and senior peers. The local guidelines on traumatic brain injuries were regarded as distinct, but it was occasionally difficult to determine when they were applicable. Mandatory admission of patients on anticoagulants was sometimes perceived as excessive. Biomarker S100b was believed to sometimes lead to delayed care.

**Conclusion:**

The participants believed that guideline adherence would increase by facilitating guideline availability, by providing concise, easy-to-understand, and well-illustrated guidelines available in printed form, as well as establishing a culture that promotes guideline use. The local guidelines for traumatic brain injuries were appreciated, but could be improved.

**Supplementary Information:**

The online version contains supplementary material available at 10.1007/s00068-021-01853-3.

## Background

Clinical guidelines are developed to help doctors make better decisions about specific situations in healthcare [1]. Guidelines are present in all areas of clinical medicine, with the objective of improving care by reducing the gap between best evidence and current practice.

However, the rate of guideline adherence is often low and varies widely [2–4]. Several barriers to guideline adherence have been identified, ranging from factors affecting individual physicians’ knowledge about and attitude towards a certain guideline to external barriers. To optimise guideline adherence, several domains need to be addressed [5].

Traumatic brain injuries (TBI) are common causes for seeking care, with potentially fatal outcomes [6]. Several guidelines have been developed and shown good validity in identifying patients at risk of significant intracranial injury, but have often been wrongfully applied [7–10]. A study conducted by our group indicated that computerised tomographies (CT) following TBIs could be reduced with strict guideline adherence [11]. Another study that we conducted regarding perceived and actual adherence to guidelines for managing patients with TBI in the emergency department (ED) showed low levels of adherence and frequent guideline misinterpretation. Efforts to improve guideline adherence were attempted, but were unsuccessful [12].

To improve guideline adherence in TBI management, general barriers to guideline adherence as well as specific shortcomings in current TBI guidelines should be identified.

## Method

The aim was to identify and explore factors that affect or are perceived to affect guideline adherence among doctors in the ED, as well as possible ways to increase guideline adherence. Secondary aims are to explore the general perception about the local TBI guidelines and their perceived deficiencies.

The ED on the study site serves approximately 250,000 inhabitants and treats approximately 60,000 patients annually.

The hospital provides local guidelines regarding the work-up and treatment of different clinical conditions. Guidelines are available through the hospitals’ Intranet, accessible on in-house computers after a mandatory log-in with personal credentials. These guidelines are written by one or a group of local or regional doctors and are generally based on national or international guidelines.

The current local guidelines for TBI management in the ED are based on the latest guidelines from the Scandinavian Neurotrauma Committee [9]. In addition to the flowchart from the original publication, the local guideline contains suggestions for the treatment of severe TBI and relevant consultant phone numbers.

To ensure adequate experience in treating patients with TBIs, a minimum of 4-week work in the section of the ED where TBI patients were managed in 2017 was required. All doctors who were eligible were identified, and 67 notices of interest were sent out (22 interns, 20 residents in emergency medicine, 16 residents in general surgery, and 9 specialists in emergency medicine) via e-mail. A personal reminder was sent when no reply was received.

Forty stated their interest in participation (13 interns, 15 residents in emergency medicine, 10 residents in general surgery, and 2 specialists in emergency medicine). Due to the low number of specialists willing to participate, both were included. Using a computerised randomisation process, five participants from each of the other categories were selected for interview. A second round of randomisation was performed, and the participants were interviewed until the predefined goal of 30 interviews was met. We tried to recruit a proportional number of participants from each category. The participants consisted of 8 (26.5%) interns, 12 (40%) residents in emergency medicine, 8 (26.5%) residents in general surgery, and 2 (7%) specialists in emergency medicine.

The predefined goal of 30 interviews was chosen, because it was deemed likely to achieve thematic saturation, based on a study by Guest et al., and would also provide adequate representation of each category of participants [13]. Saturation regarding provided answers and emerging themes was gradually reached, and a critical point was judged to be reached at around 20 interviews, where further interviews would not significantly deepen our understanding of the topic [14]. We still proceeded with the planned interviews, because they were already scheduled. No codes that were mentioned twice or more emerged after interview #26, and no codes mentioned five times or more emerged after interview #15.

Semi-structured interviews were conducted, revolving around core questions with opportunity for follow-up and branching questions [15]. Core questions were decided a priori through review of previous research, and through discussion among the authors, and were divided among four different areas (Appendix 1 See Supplementary material). A pilot interview was conducted to field-test the interview guide. During the interviews, some of the questions were rephrased to facilitate understanding and more clearly define the questions to which we sought answers.

Of the 30 interviews, 28 were transcribed and analysed. The first interview was used as a foundation for preliminary coding through a discussion with the entire research group, and the 25th interview had to be stopped midway due to an emergency call received by the interviewee, and continuation was not possible.

The first author conducted the interviews over a 3-month period. Only the interviewer and the interviewee were present, and the interview was held in a separate room at a location deemed fit by the interviewee. The interviews were recorded and transcribed by a group of professional transcribers and proofread by the interviewer. Transcripts were deidentified.

Before coding, the following six categories were created to provide a framework for organisation and subsequent thematisation:

- Internal factors that increase guideline use,
- Internal factors that decrease guideline use,
- External factors that increase guideline use,
- External factors that decrease guideline use,
- Positive aspects of the local TBI guidelines, and
- Negative aspects of the local TBI guidelines.

The first interview was coded with all the authors present, providing a common understanding of the material. The remaining interviews were coded by SV, adding codes as they emerged [16]. PAL and ML reviewed the codes and either agreed or disagreed. The rates of agreement between SV’s coding and the reviews by PAL and ML were 93.2% and 97.4%, respectively.

Three of the authors (SV, PAL, and ML) performed the thematisation of SV’s codes over the course of three sessions, which SV subsequently revised by discarding some themes and merging others. All three authors who participated in the process had to agree that a theme existed for it to be included, as well as agree that it should be excluded or merged with another theme. Eight themes were identified and broken down into several subthemes (Table 1).

**Table 1:** Themes, subthemes, number of participants, and one or more representative quotes from each subtheme

Main theme	*n*/total
**Content and presentation**	**28/28**
“There are *guidelines* that leave some room for interpretation with regards to clinical evaluation of a patient, and it can be hard for a novice doctor that hasn’t seen many such patients, to separate pathology from normal. So, the younger…or more novice, a doctor is, the more information a guideline should contain and the more experienced you are, the simpler and easier the guideline” #16—Specialist in Emergency Medicine	
“It is pretty good with flowcharts where you can see that “Yes, okay, if it gets to this I will go to this box and if gets to that I will go to that box”. I think that is pretty easy to understand” #5—Resident in Emergency Medicine	
“That there are clear rules regarding on whom the *guideline* is applicable and that it is easy to follow, that there are no uncertainties within the *guideline*” # 13—Resident in Emergency Medicine	
**Effect on care and caregivers**	**28/28**
Guidelines’ effect on patient care	18/28
“Then I think it should say a little about how to proceed and investigate. What is especially important in the anamnesis and not important in the anamnesis? Which laboratory tests should you order? Then I think it should say how to interpret different findings and how to proceed with processing, how to, for example, admit the patient or discharge with a certain medicine and at what dosages […] I have more than once come across colleagues who have only assessed the head injury and not the cervical spine” # 20—Resident in General Surgery	
Guideline use and doctors’ emotions	28/28
“What you have heard from older colleagues is that it makes you stupid, that you stop thinking a little […] You just do as the *guideline* says and sometimes you have to think outside the box. I can agree with that, but at the same time you develop *guidelines* to reduce healthcare injuries, I often think, that you miss things or that you make mistakes” # 4—Resident in Emergency Medicine	
“… Otherwise I only think it is an advantage for the patients, because it is faster, the doctor knows what to do, it is like no decision fatigue, you know exactly how to proceed, you know how to manage the patient. So, in that sense, I think it's only beneficial”. # 18—Intern	
Equality of care and resource utilisation	18/28
“Because then you know that all patients are treated in the same way. So, it is a sign… relatively safe, compared to when people read in different books and act accordingly. Then everyone gets different kind of care “ # 26—Intern	
“So, you may get a gut feeling or that you have met so many patients and the patient you have in front of you may not fit in the *guideline*. But still, according to the *guideline* you have to do… So maybe you do order a lot of unnecessary tests or examinations that you think is a little unnecessary” # 28—Resident in Emergency Medicine	
**Availability**	**28/28**
Technical conditions	28/28
“So, I think the intranet is the worst in the world. It's so… yes, but it's such a shame. There are a lot of clicks and then you often end up wrong” # 10—Intern	
“I think it would have taken a collective approach for them to be accessible easily on the intranet in some way, that they are not divided into different clinics” # 24—Resident in General Surgery	
Access time	24/28
“Then you could make a guideline book that you could have in your trouser pocket. I know, when I did my internship in Lund, there was this small guideline book, thin, which was very good” # 23—Resident in General Surgery	
“It should not be more difficult than that you have an icon on the desktop, whether it is a link or if it is a program or whatever it is, it does not matter much, but you should sort of get straight to it… it should not be more than two keystrokes away” # 3—Resident in General Surgery	
Knowledge about guidelines and where to find them	17/28
“And I think many of the employees are too poorly versed in the *guidelines*. Both doctors and other healthcare professionals about which *guidelines* we have” # 14—Resident in Emergency Medicine	
Finding the right one among many	6/28
When you check up on *guidelines*, it sometimes comes up things like “Central venous catheter”. That there can be such strange things that are not at all what you want to find. You want to get to the medically specific *guidelines*, and it is very difficult. So, there should only be one direct link there.” # 11—Intern	
**Trust in the guidelines**	**22/28**
Collective perception	13/28
“And the person who has written our local guideline is very up to date in the research, so I completely trust his judgment” # 14—Resident in Emergency Medicine	
“Because there have been some errors in our guideline book that they have had to correct afterwards. There may be on *Internedmedicin.se* as well, I know nothing about that, but there are more people who read it daily and I think it has been examined even harder because it is so publicly available” # 8—Intern	
Scientific evidence	14/28
“It is not always the case that the guideline is updated every six months and research is progressing fairly quickly in different areas. So, the absolute newest or latest recommendation for investigation and management syndrome X may not have had time to diffuse into our local guideline” # 9—Resident in Emergency Medicine	
**Workload**	**27/28**
Subjective stress	15/28
“When you get stressed you become a little more primitive, you go into your inner self, you solve problems, and you do what you think is best and go more on gut feeling” # 21—Intern	
“I almost think that it *[stress]* makes you use it *[guidelines]* more, because if there is a lot that bothers and interrupt you think a little worse” # 4—Resident in Emergency Medicine	
Work environment	15/28
“It sometimes happens, when there are a lot of patients that seek care, that we can get a shortage of nurses who can take this test which has a limit of six hours. I would probably say that it may be a reason to do a CT then because we also have a lack of beds, we cannot admit and observe them for twelve hours in a simple way” # 16—Specialist in Emergency Medicine	
Amount of time at disposal	13/28
“No, I do not sit and look for a *guideline* that I cannot find in half an hour, instead I can take the next patient” # 15—Resident in Emergency Medicine	
**Culture**	**28/28**
Attitude among staff	25/28
“The nurses are well acquainted with it, so often even before we meet the patient, they have taken a history of the patient and asked how long it has been since the head injury and if they are on anticoagulation. And then they come and ask us if we want to order an S100B” # 14—Resident in Emergency Medicine	
“The nurses or assistant nurses believe that the patients should be managed in a certain way. They often want things to flow on and go fast and so on” # 12—Resident in General Surgery	
Leadership	23/28
“On the other hand, it has happened that I have wanted to treat the patient according to our local guideline, but the person I consult with wants to do something else. But then it’s on them” # 7—Intern	
**Patient-related factors**	**24/28**
Incompatibility between patient and guideline	20/28
“So, it is me who sees the patient in front of me, it's not our *guideline* that sees it. So, if I think it's wrong, I’ll do it my way.” # 15—Resident in Emergency Medicine	
Complexity in the individual case	11/28
“Then there are always patients who are… it is difficult to really put your finger on what is the cause of seeking care or what is the triggering problem and then you cannot turn to specific guidelines, because… Yes, if the symptoms go together and it is not really clear, then you do not know which guideline to choose “ # 7—Intern	
“While a *local guideline* is often based on the patient having a medical condition. But if the patient has more than one condition that you have to weigh in, you may sometimes need to make an intermediate solution, which means that you have not fully followed *the local guideline*, which can sometimes make it a little difficult. “ # 9—Resident in Emergency Medicine	
**Doctor-related factors**	**23/28**
Individual doctor competence	13/28
“… There are those who temporarily work at the clinic who may need a *local guideline* and also older colleagues who have not encountered this for a long time, or… yes, also need a little… go back and check” # 11—Intern	
Experience	13/28
“Less experienced, new doctors, I think use *local guidelines* in the best possible way. But an older experienced person, a doctor, is probably able to tailor his patient management better than a *local guideline*” # 29—Resident in Emergency Medicine	
Convenience and ego	8/28
“If I have received a positive that I should x-ray the neck and the patient has a small cut in the forehead, then I usually have a fairly low threshold to X-ray the head as well” # 9—Resident in Emergency Medicine	
“If you feel that you would not want to follow it *[local guideline]*, there can be quite a lot of problems if a problem or complication should arise later. And if you have not followed the *local guideline*, many will be afraid of it as well” # 28—Resident in Emergency Medicine	

Representative quotes were selected to convey the essence of each subtheme (Table 1). Quotes regarding circumstances unique to the study site were in some cases slightly rephrased to facilitate understanding without changing the underlying meaning. These cases can be identified by the use of italic font.

## Results

Twenty-six (92.8%) of the interviewees had a positive attitude towards guidelines, especially if the user was inexperienced. Two remaining participants were neutral towards guidelines, emphasising the risk that guidelines might impair tailoring of treatment to the individual patient. Twenty-one (75%) of the interviewees had either accessed or possessed knowledge about the local TBI guidelines.

### Content and presentation

Twenty participants said that it was important for a guideline to be concise. Nine participants brought up “too much non-essential information” as an obstacle to use, since excessive amount of text increased the likelihood of missing vital information. At the same time, 11 participants brought up the presence of disease-specific background information as a positive factor.

Twenty participants believed that visual aids, such as pictures and flowcharts, could increase usability and provide sequential treatment schemes that would be easy-to-understand.

Seventeen participants stressed the importance of arranging different sections of the guidelines in a logical order. Three of these participants and six others also specified the need to be able to overview the guidelines at a quick glance, with easily understood and relevant headings.

Fourteen participants mentioned clear recommendations that were unlikely to be misinterpreted as important. Five of these participants and four others also mentioned that it was important for the guidelines to clearly state under what circumstances and in which patients they would be applicable.

### Effects on care and caregivers

#### Guidelines’ effect on patient care

Sixteen participants either mentioned that guidelines provided guidance or hints in their patient management or that they viewed this as an important function of a guideline. Five of these participants plus another one also stated the importance of a guideline providing guidance regarding management or follow-up after the emergency had been resolved. Two participants were also in favour of reminders to evaluate other conditions that frequently co-exist with the primarily managed condition.

#### Guideline use and doctors’ emotions

Twenty participants felt more secure in their decisions when they had guidelines to lean on. Twelve participants believed that the use of guidelines could lead to premature diagnostic closure or that doctors could become too rigid in situations where more individualised management would be beneficial. Of these participants, nine still thought that using guidelines led to safer patient management, and five still believed that guidelines provided “the correct way” of managing a condition.

Fifteen participants stated that using guidelines could also make patient management more efficient. Six of these participants plus another one believed that using guidelines led to less decision fatigue.

#### Equality of care and resource utilisation

Sixteen participants brought up guideline use as positive in terms of ensuring that each patient would be evaluated and managed in a similar fashion.

Five participants believed that using guidelines led to better utilisation of resources and reduced unnecessary tests, but four other participants thought that strict guideline adherence could increase resource use.

### Availability

#### Technical conditions

All participants brought up at least one technical issue as a barrier to guideline use. The main concern, mentioned by 22 participants, was the difficulty in finding the guidelines on the Intranet. The retrieval process was described as time-consuming and cumbersome by 13 of these participants and by an additional six participants who did not express difficulty in finding the guidelines. Nine of these 22 participants indicated that this difficulty could partly be attributed to the layout of the intranet.

The guidelines were placed in different locations, depending on which department wrote them. Eleven participants considered it important for guidelines relevant for managing a wide variety of conditions to be in the same place.

Other negative factors were the need for a sign-in to access the guidelines, long loading times, old or corrupted documents, and frequent reorganisations of the Intranet.

#### Access time

Twenty participants believed that printed guideline documents available on site could increase guideline use.

Seventeen participants wished for an improved search function on the Intranet, and 12 participants wanted a direct link from the Intranet home page and the desktop computers to all available guidelines.

#### Knowledge about guidelines and where to find them

Fourteen participants stated the importance of knowing that guidelines exist. Six of these participants and two others also brought up the fact that a potential user had to know their specific location.

#### Finding the right one among many

Finding a specific guideline was made difficult by both the sheer number of available guidelines (according to two participants) and the abundance of non-medical guidelines that cluttered the web page (according to four participants).

### Trust in the guidelines

#### Collective perception

According to nine participants, the knowledge that a set of guidelines was developed through a thorough process by people competent to do so was an important factor in them trusting the guidelines.

Five participants deemed national or publicly available guidelines more reliable, lowering the risk of outdated or inaccurate science behind the claims.

#### Scientific evidence

Thirteen participants brought up scientific background as an important factor for trust in a guideline. Eight participants said that it was important for a guideline to be based on validated science, and some mentioned that a presentation of references was a positive factor.

Eight participants brought up an insufficient scientific background and the belief that the recommendations in a set of guidelines were outdated and not based on the latest research as a factor that decreased their trust in a guideline.

### Workload

#### Subjective stress

Twelve participants believed that feeling stressed could negatively affect the willingness to use guidelines. This was most frequently attributed to the cumbersome and time-consuming process of locating and accessing the guidelines, and many preferred to consult a more senior colleague instead when stressed.

Four other participants believed that elevated levels of stress could lead to increased guideline use by making it easier to sort one’s own thoughts and prevent missing important steps.

#### Work environment

Thirteen participants cited ED crowding and high in-hospital bed occupancy as factors that occasionally made them disregard a guideline. Regarding patients treated with anticoagulants, the recommendation for their admission for 24-h observation after TBIs was frequently mentioned as not followed in situations of high levels of bed occupancy. Five participants also believed that guideline adherence was negatively affected by the shortage of nursing staff.

#### Amount of time at disposal

Thirteen participants brought up the lack of time to retrieve, read, and apply guidelines as a factor that decreased guideline use. This factor was closely related to factors contributing to subjective stress but differed in being mainly an issue of too much to do in too little time, while the stress factor was focused on the doctor’s reaction to this lack of time.

### Culture

#### Attitudes among staff

Twenty-four participants experienced using guidelines when working in the ED as part of the norm, and they had either been encouraged to use a certain guideline or witnessed their peers using it.

Seven participants emphasised including all staff groups when developing a guideline. Particularly, nurses are heavily engaged in the initial care, and they are directly involved in certain time-sensitive interventions or examinations. The participants believed that keeping nurses updated on the recommended management could increase guideline adherence.

At the same time, two participants brought up negative peer pressure from members of other professions as a factor that might lead to lower guideline adherence, especially if following a guideline would cause more work than bypassing the guideline.

#### Leadership

Eighteen participants had either been encouraged to use a certain guideline by their superiors or they believed that encouragement from superiors would increase adherence. Four of these participants and five others had disregarded a guideline based on recommendations from their superiors.

### Patient-related factors

#### Incompatibility between patient and guideline

Ten participants said that if they believed that patient management, as stated in the guidelines, would not benefit the patient, they would not use the guidelines. Citing examples, they believed that the guidelines underestimated the severity of the patient’s condition, the patient had too many co-morbidities that would make him/her ineligible for a certain treatment no matter what the examinations showed, or the patient’s condition was deemed benign and not requiring treatment.

#### Complexity in the individual case

High complexity cases could hinder guideline application, according to eight participants. It might be difficult to place a patient under a certain category of a guideline, or the patient might present with a mix of symptoms that would make it hard to know which guideline to apply.

Five participants said that treating patients with multiple ailments could lead to lower guideline adherence. The recommendations provided in one guideline would sometimes contradict those of another guideline.

### Doctor-related factors

#### Individual doctor competence

Eleven participants stated that if a doctor perceived oneself as less competent in a certain area, this could lead to a higher tendency to use guidelines. According to four participants, guideline use could also increase if the doctor believed that it was a good way to stay updated about current recommendations.

#### Experience

Nine participants believed that a more experienced doctor would have a better ability to tailor work-up and treatment to the individual patient than a guideline. They also believed that more experienced doctors tended to stray from guideline recommendations. Three of these participants and two others also believed that previous clinical experience in managing a certain condition could lead to a lower tendency to search for and apply a guideline, since these doctors would already have established a satisfactory routine.

#### Convenience and ego

Six participants sometimes strayed from guideline recommendations regarding which radiological evaluations to order. The most frequently cited example was to include a CT scan of the head, even though it might be unnecessary, when the patient had already qualified for a CT scan of the cervical spine.

Two participants also viewed adhering to a guideline as a means to cover one’s back in case of an adverse event. Two other participants believed that social pressure and fear of being perceived as bothersome when requesting certain procedures or tests could decrease guideline adherence.

## Thoughts on the local TBI guidelines

Nineteen participants viewed the local TBI guidelines as distinct. Ten participants experienced uncertainty about what level of severity of trauma was required for inclusion in the TBI treatment regimen. A common situation was a patient presenting with a seemingly trivial injury but co-existing factors that would mandate a more thorough work-up, such as the use of anticoagulants.

“There is nothing about how severe the head trauma needs to be. If you walk into a lamppost, does it count as a sufficiently serious trauma to justify a CT just because you are over 65 and are under treatment with a platelet aggregation inhibitor?”

#2—Resident in General Surgery.

Four participants believed that the current recommendation about mandatory hospital admission of patients on anticoagulants and with a normal CT scan of the head was excessive.

“The care might be a bit exaggerated, especially for those with minimal head injury and ongoing anticoagulation. If you then have to follow it [local guideline], it will be a lot of CT, a lot of hospitalisation and a lot of care.”

#13—Resident in Emergency Medicine.

The use of biomarker S100B in the context of the guidelines was sometimes perceived as problematic. Examples of this were false-positive results leading to unnecessary CT scans and longer ED stays due to long analysis times.

“And you order an S100B that is false positive, which when I feel can sometimes force you to do a CT on a patient when you really wanted to not do it, if it is a young patient, for example.”

#14—Resident in Emergency Medicine.

Some participants believed that key patient groups were missed in the current guidelines, such as patients presenting after 24 h and under treatment with multiple platelet inhibitors. A few participants were also uncertain about how to manage intoxicated patients. The distinctions among different degrees of head injury were sometimes a bit unclear and could be more visually emphasised.

“I think if I had done it, I would have made larger letters or capital letters where it says acetylsalicylic acid or NOAC, so that when you look at it quickly it is clear that ‘Oh, here it was something different. Those patients should go into another category’.”

#9—Resident in Emergency Medicine.

## Discussion

Guidelines have been a part of medical practice for many decades, with obstacles present from the start. Cabana et al. identified several barriers to guideline adherence in the domains of doctor knowledge (lack of familiarity or awareness), attitudes (lack of agreement, of outcome expectancy, of self-efficacy or of motivation), and behaviour (external barriers, guideline factors, or environmental factors) [5].

Many of these barriers could be identified in our materials, but our barriers were more centred on various aspects of availability. These were often related to the difficulty in finding the guidelines, a cumbersome retrieval process, and lengthy documents. Stiell et al. received similar answers regarding ED overcrowding that required more expedient care, possibly favouring a quicker but not entirely guideline-adherent approach [8]. An implementation study for the National Institute for Health and Care Excellence 2014 head injury guidelines showed a higher than expected percentage of early discharges from the ED, and the authors speculated that this could, at least partly, be attributed to ED overcrowding [17].

The most frequently mentioned factors that positively affected guideline use was a short document, appropriate visual aids, easily accessible as either a printed document or on a computer, and a culture among peers and management that promotes guideline use. A guideline that provides simple, easy-to-understand recommendations can intuitively be regarded as a factor that would allow higher levels of adherence, which is supported by previous studies in other settings and regarding other conditions [18, 19]. The presence of guidelines in the work environment as a reminder of their existence and their intrinsic characteristics is also a factor that previous studies have either shown or speculated as actually or potentially beneficial to increase use. A workplace culture that emphasises guideline adherence through colleague encouragement and endorsement from the department management can also increase guideline adherence [17–19].

Given the wide spectrum of potential barriers to adherence, a broad array of interventions targeting these different barriers should be sought. Based on our results, we recommend the following interventions for increasing guideline use in the ED:

1. Increase availability of physical documents. Provide the opportunity for everyone to get a printed guideline pamphlet that fits in one’s pocket.
2. Provide a direct link on the desktop of the computers to all guidelines relevant to doctors in the ED, which should be logically sorted.
3. Reduce guideline length to the minimum. Provide supplementary materials that include background information about the diagnoses and references when relevant.
4. Provide well-designed and intuitive figures and flowcharts when appropriate.
5. Provide guidelines on both the diagnostic work-up of presenting symptoms and the management of specific conditions.
6. Design guidelines with their intended users and their clinical situations in mind. Disseminate the guidelines to other staff groups involved in patient care.
7. Launch a rigorous information campaign regarding the existence and location of the guidelines, aimed at specialists and residents who in turn influence more junior colleagues. A local culture of guideline adherence would further reinforce their use.
8. Keep guidelines updated. Designate a person, preferably with a scientific background that is respected among peers, to update the guidelines at regular intervals and keep others informed of these updates.

De Wit et al. prepared a list of ten recommendations for implementing changes in practice based on a systematic review and an online survey involving Canadian emergency physicians [20]. While many of our provided recommendations correspond to theirs, we put more emphasis on early involvement of the doctors who were the intended users. This was done to ensure that they understood the current evidence and were aware of the present gap in practice, as well as the importance of focusing on changing practice where care could be significantly improved. The impacts of these practice changes should be measured whenever possible, regarding both compliance and patient-centred outcomes. To ensure sustained adherence to new practice changes, a business plan that provides adequate personnel resources with clearly defined longitudinal roles should be established.

Although many of the emerging themes are represented in previous research, our study has limitations. This was a single centre study interviewing a limited number of doctors on a specific issue, and our findings should be viewed as hypothesis generating. The relative importance of each identified factor needs to be studied prospectively.

Compared with interviews asking fixed questions, semi-structured interviews provide an opportunity for deeper penetration of the research topic, as well as the risk of bias. In this case, the interviewer was a practicing intern with previous extensive knowledge of the topic at hand. Branching questions that were asked might have been subject to both confirmation and question-order bias. Many of the participants were acquainted with and sometimes even colleagues of the interviewer. Everyone was to some extent aware of the interviewer’s engagement in promoting and improving the local TBI guidelines, and some degree of social pressure was possibly present. This could make them state a higher level of guideline adherence and may be a more positive attitude towards the local TBI guidelines.

## Conclusion

The participants believed that guideline adherence would increase by facilitating guideline availability, by providing concise, easy-to-understand, and well-illustrated guidelines available in printed form, as well as establishing a culture that promotes guideline use. The local TBI guidelines were appreciated but could be improved.

## Supplementary Information

Below is the link to the electronic supplementary material.

Supplementary file 1 (PDF 57 KB)

## Data Availability

The datasets used and analysed during the current study are available from the corresponding author on reasonable request.

## Abbreviations

CT: Computerised tomography
ED: Emergency department
NOAC: Novel oral anticoagulants
TBI: Traumatic brain injury
