# Supplementary material for: Ways to improve guideline adherence in the emergency department: an interview study on the management of traumatic brain injuries
Source: Eur J Trauma Emerg Surg. 2022 Feb 3;48(6):4499–508. doi: 10.1007/s00068-021-01853-3 (PMC9712355; doi:10.1007/s00068-021-01853-3)
Supplement: Supplementary file 1 — (PDF 57 KB) [file 68_2021_1853_MOESM1_ESM.pdf]

Interview template

Theme 1: General

1. Tell me what a local guideline is, how it is produced, and at what levels within the healthcare organization that a local guideline can be produced and exist.
2. What is your personal opinion on local guidelines in general?
3. What do you mean when you say you use or do not use local guidelines?
4. What do you feel is important regarding the design and structure of local guidelines?
5. Do you feel that you have the ability to interpret and apply the local guidelines you have used? If so, does this apply to any specific local guideline?
6. How do you think the use of local guidelines in general affects the quality of care that the patient receives?
7. Do you usually use local guidelines or other clinical management guidelines in your work? If not, why not? If so, why not?

Theme 2: Head injuries

8. Do we currently have a local guideline for adult patients with head injuries?
9. Have you used this local guideline in your work?
10. How is this structured in terms of design and application? Describe it to me.
11. How do you experience working based on the current local guideline for head injuries?
    - Do you experience any clear disadvantages with this local guideline?
    - Do you feel that some special patient groups are repeatedly difficult to apply this local guideline to?
    - How much background knowledge would you say you have regarding the recommendation/guideline on which our current local guideline is based?
    - How do you think this local guideline affects your work with this patient group?
    - Do you feel that patients are treated differently from what you thought if you follow this local guideline?

12. Have you used other, published guidelines or decision support for the treatment of patients with head injuries?
    - Where did you find these guidelines?
    - How do you experience working from these guidelines?
    - Do you trust these guidelines?
    - How much knowledge do you have about where these guidelines come from and on what evidence they are based on?

13. Can you see any risks for patients to indiscriminately use local guidelines or other guidelines? Is this something you have thought about in your everyday clinical practice?
14. How do you think the use of our head injury local guideline affects the quality of the care the patient receives?
15. How do you handle head injury patients in practice at the moment?

Theme 3: External factors

16. Local guidelines are currently only available via the intranet. Have you ever retrieved a local guideline from the intranet?
17. How do you experience the process of retrieving these local guidelines from the intranet?
18. Has any colleague tried to influence you to use / not use local guidelines? If yes, describe.
19. Has any manager or other superior tried to influence you to use / not use local guidelines?
20. How would you say that a perceived lack of time in the emergency department affects your use of local guidelines? Can you imagine that this also applies to others?
21. How would you say that subjective stress at work affects your use of local guidelines? Can you imagine that this also applies to others in the same situation?
22. Do you feel that the hospital/emergency department has adequate resources for you to be able to follow the local guideline as it is written?
23. Are there other factors in the work environment in the emergency room that affect local guideline use?
24. Are there any situations that you imagine that make you deviate from local guidelines more often?

Theme 4: Own reflections

25. What factors make the use of local guidelines in the emergency department more difficult? Does this apply to all local guidelines or only local guidelines for head injuries?

26. How do you think the use of local guidelines can be increased in the emergency department? Does this apply to all local guidelines or only local guidelines for head injuries?
